# Long bone deformity correction and bone lengthening procedures

**DOI:** 10.1007/s11832-016-0796-7

**Published:** 2016-12-08

**Authors:** Pierre Lascombes, Hakan Omeroglu

**Affiliations:** 1 Division of Paediatric Orthopaedics, Hôpitaux Universitaires de Genève, Rue Willy Donzé 6, 1211 Geneva 14, Switzerland; 2 Department of Orthopaedics and Traumatology, Faculty of Medicine, TOBB Economy and Technology University, 06530 Ankara, Turkey

Last year’s 34th Annual Meeting of the European Paediatric Orthopaedic Society (EPOS) was held in Marseille, France, on 15–18 April. A comprehensive pre-meeting course held on 15 April was dedicated to many complex aspects of long-bone deformity correction and bone-lengthening procedures. The present issue of the* Journal of Children’s Orthopaedics* collates manuscripts of the presentations made at that advanced course, and thus provides a summary of the latest strategies for and descriptions of most of the more difficult approaches to long-bone correction and lengthening. References to some additional recent works in this field have also been added to the reference lists originally provided with the manuscripts.

Guided growth is one of the most common surgeries used to treat such conditions. The first part of this issue reports on the mechanism and reversibility of growth modulation in deformity corrections, as well as the role of growth modulation in limb lengthening. During the course, Elhanan Bar-On described guided femoral rotational growth in an animal model [1]. New perspectives and applications will surely be developed in the future [2–5].

The second part of this issue is dedicated to long-bone lengthening procedures and updated techniques. Among external fixators, the classic Ilizarov fixator is increasingly being replaced by hexapod fixators, as these allow easier 3-D correction through the use of modern computer software. The application of additional flexible intramedullary nails leads to an average decrease in the healing index of 20%. Complications are also less likely with external fixation, as the fixator is removed earlier. The development of motorized and magnetic intramedullary lengthening nails is also contributing to improvements in lengthening reliability and quality. Two other subjects of debate are the indications for a bridging joint, soft-tissue release, and physiotherapy, and the problem of an unstable knee in congenital lower-limb deficiency.

With regards to surgical technique, the third part of this issue concerns the master techniques for tibial and fibular hemimelias, as well as for humerus and forearm lengthening, including in cases with multiple exostoses [6]. Both of the manuscripts on tibial and fibular hemimelias should be considered state-of-the-art papers, and they provide definitive guidance for any surgeon who needs to treat these congenital abnormalities.

The final part of this issue covers the controversial topic of bilateral lengthening. Approaches to achondroplasia differ from those used in cosmetic indications. During the pre-meeting course, Dror Paley presented his experiences of cosmetic lengthening with magnetic nails [7], and Francesco Guerrischi described his with external fixators. Another important topic is the prevention of pin-tract infections during treatment. The growth remaining after the lengthening procedure must be perfectly understood in order to ensure that maximum natural growth is achieved, even in cases with multiple lengthening during childhood. Finally, amputation and rotationplasty may still be indicated for severe limb deficiencies [8].

We send our warmest thanks to our hosts in Marseille, Gérard Bollini, and Jean-Luc Jouve, who were largely responsible for the course’s success, as well as to all the authors who agreed to provide the updated and concise manuscripts which document their work and are included in this exceptional issue of JCO.
